# ILC2s Induce Adaptive Th2-Type Immunity in Acute Exacerbation of Chronic Obstructive Pulmonary Disease

**DOI:** 10.1155/2019/3140183

**Published:** 2019-06-20

**Authors:** Min Jiang, Huifang Liu, Zheng Li, Jing Wang, Fengbo Zhang, Kaixiu Cao, Fengsen Li, Jianbing Ding

**Affiliations:** ^1^Xinjiang Laboratory of Respiratory Disease Research, Traditional Chinese Medicine Hospital Affiliated to Xinjiang Medical University, Urumqi 830011, China; ^2^Department of Clinical Laboratory, First Affiliated Hospital of Xinjiang Medical University, Urumqi 830054, China; ^3^Department of Immunology, College of Basic Medicine, Xinjiang Medical University, Urumqi 830011, China

## Abstract

To investigate the effect of ILC2s on Th2-type adaptive immunity during the acute exacerbation of chronic obstructive pulmonary disease (AECOPD), the study enrolled healthy people, stable COPD patients, and AECOPD patients. Flow cytometry was used to detect Th1, Th2, and ILC2 in the peripheral blood and CD80 and MHC II levels on ILC2. The mRNA levels of *GATA3*, *RORα*, and *CRTH2* of ILC2s were detected by RT-PCR. In addition, ILC2s from the peripheral blood of AECOPD patients were cocultured with CD4^+^ T cells from the peripheral blood of healthy controls. Cytokine levels in serum of the three groups and the in vitro coculture supernatants were measured by ELISA. Compared with the stable COPD group or the healthy control group, Th2 in the peripheral blood of AECOPD group increased dramatically, inducing an increase of Th2/Th1 ratio in AECOPD patients. Meanwhile, the level of IL-4 in the serum of this group was also increased. However, we also detected ILC2s in the peripheral blood of the AECOPD group and found that it was also increased, alone with the increased *GATA3*, *RORα*, and *CRTH2* mRNA levels. We also found that the CD80 and MHC II on ILC2 were significantly upregulated and the proportion of MHC II^+^ ILC2 cells was significantly positively correlated with the proportion of Th2 cells in AECOPD patients. To further demonstrate the effect of ILC2 on Th2 cells, we cocultured ILC2 with CD4^+^ T cells in vitro, which also showed a significant increase of Th2 ratio as well as Th2-associated cytokines IL-4, IL-5, and IL-13. However, we found that this effect of ILC2s on Th2 cells could be inhibited by the addition of anti-MHC II. The Th2/Th1 balance shifts to Th2 in AECOPD. ILC2s may function as APC by the upregulation of MHC II and regulate adaptive immunity shift to Th2-type response in AECOPD.

## 1. Introduction

Chronic obstructive pulmonary disease (COPD) is a chronic inflammatory disease of the respiratory tract characterized by persistent incomplete reversible airflow limitation [1, 2]. COPD can develop into acute exacerbation of COPD (AECOPD), featured by symptoms of cough, phlegm, asthma, and dyspnea. In AECOPD, the lung function of patients is rapidly deteriorated, accompanied by inflammation and fever. Recurrent AECOPD is an important factor in promoting the progression of COPD, leading to a decline in the quality of life of patients and an increase in mortality [3–6]. Lower respiratory tract bacterial infection is an important factor in the occurrence of AECOPD, which can lead to immune dysfunction. Studies also found that the abnormality of T cell subsets is the main cause of immune dysfunction in COPD [7–9] and Th1/Th2 imbalance may be one of the causes of AECOPD [10]. It is reported that the activation and proliferation of Th2 cells in the peripheral blood of patients with AECOPD were more obvious, resulting in a significant downregulation of Th1/Th2 ratio and a shift to Th2 responses [11]. However, the cause of the shift of the adaptive immunity to the Th2-type immune response in the AECOPD remains unclear.

The type 2 innate lymphoid cells (ILC2s) are the main effecter cells of early Th2-type immune response and play an important role in inducing adaptive Th2-type immune response [12, 13]. ILC2s can rapidly secrete Th2-type cytokines such as IL-5 and IL-13 under the stimulation of intrinsic cytokines such as IL-33 and IL-25, thereby regulating the innate immune system and the adaptive immune system to participate in Th2-type immune responses [14, 15]. Previous study has shown that professional antigen-presenting dendritic cells are essential for inducing Th2-type immune responses by expressing MHC class II molecules [16]. Other cells such as B cells, eosinophils, and ILC2s can also express MHC class II molecules to function as antigen-presenting cells (APC) [17, 18]. In addition, ILC2s highly express OX40L, ICOS, and ICOSL and costimulatory molecules of CD80 and CD86, which can regulate the differentiation of CD4^+^ T cells [19, 20]. Pulmonary inflammation in COPD is characterized by both innate and adaptive immune responses, and ILC2s are involved in the innate immune response [21]. However, the role of ILC2 in regulating Th2-type responses in COPD remains unclear.

In this study, we investigated the effect of ILC2s on Th2-type adaptive immunity during AECOPD. We found that ILC2 could play as APC by upregulating CD80 and MHC II molecules, thereby regulating Th2-type immune response in AECOPD.

## 2. Materials and Methods

### 2.1. Patients

We included outpatients and inpatients from November 2017 to November 2018 in Xinjiang Uygur Autonomous Region Chinese Medicine Hospital, all of whom met the diagnostic criteria for COPD. Among them, 98 patients (59 males and 39 females) were with AECOPD, with an average age of 67 ± 8.5 years. There were 112 patients (68 males and 44 females) with stable COPD, with an average age of 67 ± 8.2 years. We also enrolled 110 healthy controls (69 males and 41 females) with an average age of 66 ± 6.9 years. The demographic information and medical history of patients were recorded (Table 1), and all the patients were scored using the COPD Assessment Test (CAT) Scale and the Modified Medical Research Council (mMRC) Dyspnea Scale. The six-minute walk distance (6MWD) was also measured. Informed consent was obtained from each patient. The project was approved by the Ethics Committee of Xinjiang Uygur Autonomous Region Chinese Medicine Hospital.

### 2.2. Diagnosis Inclusion and Exclusion Criteria

COPD and AECOPD diagnostic criteria were in line with the Global Strategy for the Diagnosis, Management, and Prevention of Chronic Obstructive Pulmonary Disease (2011 Revision), published jointly by the American Thoracic Society and the European Respiratory Society [22]. Inclusion criteria for COPD were as follows: (1) patients with COPD diagnosed based on clinical manifestations (such as chronic cough, cough, and/or dyspnea), exposure history of risk factors, physical signs, and pulmonary function tests; (2) patients with incomplete reversible airflow limitation (FEV1/FVC < 70% after bronchodilator). At the same time, the AECOPD group also met the following criteria: COPD patients had cough in a short term and the cough was aggravated accompanied by purulent sputum, asthma, dyspnea, and fever. Exclusion criteria were as follows: (1) patients with other respiratory diseases or other systemic infections; (2) patients with serious primary diseases such as severe cardiovascular diseases, hepatorenal diseases, and hematopoietic system diseases; and (3) patients who used antibiotics, systemic glucocorticoids, or immunosuppressants within 30 days before enrolment. All the healthy controls were of neither symptom of infection nor abnormal lung function.

### 2.3. Sampling

The peripheral blood was collected before antibiotic or hormone therapy. PBMCs were isolated from the whole blood by Ficoll density gradient method. The whole blood was collected from all subjects and then placed at room temperature for 30 min. Then, the serum was separated by centrifugation at 3000 rpm for 10 min, and the serum was stored at -80°C until use.

### 2.4. Flow Cytometry

For detection of Th1 and Th2, 200 *μ*L of peripheral blood was incubated with an equal volume of RPMI-1640 medium along with 2 *μ*L Leukocyte Activation Cocktail, with BD GolgiPlug (550583, BD, San Jose, CA, USA) at 37°C, 5% CO_2_ for 6 h. Then, the cells were stained with APC-Cy7-CD3 (SK7, BD, San Jose, CA, USA) and FITC-CD4 (RPA-T4, BD, San Jose, CA, USA) for 30 min at room temperature. After that, cells were fixed and permeabilized at 4°C for 40-50 minutes in the dark. After washing, the cells were stained with APC-IFN-*γ* (B27, BD, San Jose, CA, USA), PE-IL-4 (8D4-8, BD, San Jose, CA, USA) at 4°C for 40-50 min in the dark. Then, the percentage of CD3^+^CD4^+^IFN-*γ*^+^ T cells and CD3^+^CD4^+^IL-4^+^ T cells was measured by flow cytometry.

For detection of CD80/MHC II of ILC2, 200 *μ*L of peripheral blood was incubated with the following antibodies (all from BD unless mentioned), including Lineage-FITC, CD3 (UCHT1), CD19 (HIB19), CD123 (7G3), CD11b (M1/70), CD11c (B-ly6), CD8 (RPA-T8), Fc*ε*RI (AER-37 (CRA-1)), CD14 (M5E2), CD4 (RPA-T4), CD56 (B159), CD45-APC-Cy7 (2D1), CRTH2-PerCP-Cy5.5 (BM16), CD127-PE-Cy7 (HIL-7R-M21), HLA-DR-APC (G46-6), and PE-CD80 (L307.4) at room temperature for 30 min in the dark. The cells were lysed using red blood cell lysate for 10-15 min. After washing, the cells were resuspended, and the percentage of Lin^−^CD45^+^CD127^+^CRTH2^+^ cells and Lin^−^CD45^+^CD127^+^CRTH2^+^HLA-DR^+^ and Lin^−^CD45^+^CD127^+^CRTH2^+^CD80^+^ cells was detected by flow cytometry.

All flow analyses were performed using the LSR II flow cytometer (BD, USA) and analyzed using the Kaluza software (Beckman Coulter Inc.).

### 2.5. Cell Sorting

Peripheral blood of the healthy control group, the stable COPD group, and the AECOPD group was collected, and ILC2s from each group were then sorted by magnetic beads (STEMCELL, Vancouver, BC, Canada). The CD4^+^ T cells were sorted from the peripheral blood of healthy controls with magnetic beads (Miltenyi Biotec, Bergisch Gladbach, Germany).

### 2.6. Real-Time PCR

Total RNA was extracted from ILC2s using TRIzol LS (Takara, Tokyo, Japan). Then, the cDNA was synthesized from the isolated total RNA using Transcriptor first strand cDNA synthesis kit (Takara, Tokyo, Japan). The mRNA levels of *CRTH2*, *GATA3*, and *RORα* were detected with real-time PCR by using BIO-RAD CFX96 detection system. Primers for these genes are listed in Table 2. The PCR procedure was as follows: 95°C for 5 min, followed by 40 cycles of 95°C for 30 s and 60°C for 30 s. The expression levels were calculated with 2^-△△CT^ method and normalized to GAPDH.

### 2.7. Coculture

The sorted ILC2s from AECOPD patients were seeded at 500 cells/well in 96-well plates and cultured with incomplete IMDM medium supplemented with 500 ng/mL IL-2 (200-02-10, PeproTech, Rocky Hill, NJ, USA), 500 ng/mL IL-25 (8134-IL-025, R&D, Minneapolis, MN, USA), 500 ng/mL IL-33 (R&D, Minneapolis, MN, USA), and 500 ng/mL TSLP (300-62-10, PeproTech, Rocky Hill, NJ, USA) for 5 days. The CD4^+^ T cells sorted from healthy controls were then seeded at 1 × 10^5^/well in 96-well plates.

ILC2 and CD4^+^ T cells were then cocultured at 1 : 1 ratio and were divided into coculture group, anti-MHC class II mAb (20 mg/mL, eBioscience) blocking group, and anti-MHC isotype (eBioscience) control group. The cells were incubated at 37°C, 5% CO_2_ for 4 days. After that, the culture supernatant was collected. Then, the CD3^+^CD4^+^IL-4^+^ cells were analyzed by flow cytometry.

### 2.8. ELISA

Levels of IL-5, IL-13, IL-4, and IFN-*γ* in serum and the coculture supernatant were measured by double antibody sandwich enzyme-linked immunosorbent assay (ELISA) kits. All ELISA kits were purchased from Hangzhou Lianke Biotechnology Co. Ltd. (Hangzhou, China). Briefly, an ELISA plate was coated with capture antibody. The serum or sample supernatant was then added, and horseradish peroxidase-conjugated secondary monoclonal antibody was added. After washing with PBST three times, tetramethylbenzidine was added in the dark for colour development. Absorbance was determined at 450 nm and a reference wavelength of 570 nm using a spectrophotometer. Concentrations were calculated based on a standard curve.

### 2.9. Statistical Analysis

Statistical analysis was performed using GraphPad Prism 5 (GraphPad Software Inc., San Diego, CA, USA). Measurement data are expressed as mean ± standard deviation (SD). One-way ANOVA and Mann-Whitney *U* test were used to compare the data among multiple groups. The *t-*test was used for the comparison between two groups. Count data were expressed as percentages and analyzed by *χ*^2^ test. Pearson correlation analysis was used for correlation analysis. *P* < 0.05 means statistical difference and *P* < 0.01 means that the difference is extremely significant.

## 3. Results

### 3.1. Clinical Data of Patients

A summary of patient demographics is shown in Table 1. The body mass index (BMI) was 24.5 ± 3.9 kg/m^2^ in the AECOPD group, 25.1 ± 3.4 kg/m^2^ in the stable COPD group, and 24.75 ± 2.76 kg/m^2^ in HCs. No difference of demographic characteristics was found among groups. Forced expiratory volume in one second FEV1/forced vital capacity (FVC) of AECOPD patients was obviously lower than that in stable COPD patients and HCs (all *P* < 0.001). In addition, FEV1% and FVC% of AECOPD patients were also obviously lower than those in the other two groups. Compared with healthy controls, the mMRC score and CAT results of AECOPD patients were significantly increased (*P* < 0.05). Besides, IL-4 and IFN-*γ* expression in AECOPD patients was markedly higher than that in stable COPD patients (*P* < 0.05).

### 3.2. Th2/Th1 Balance Shifts to Th2 in AECOPD

**Figure 1:**
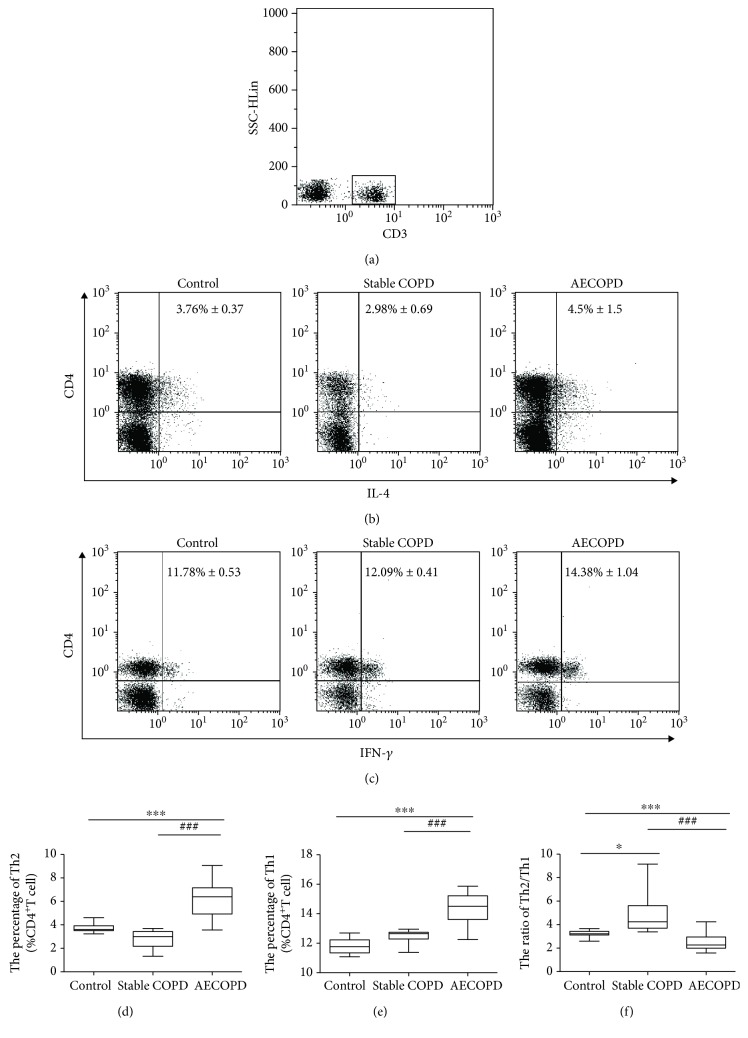
Percentage of Th2 cells and Th1 cells in the peripheral blood of three groups. Th2 cells and Th1 cells from healthy control, stable COPD, and AECOPD groups were analyzed by flow cytometry. (a) CD3^+^ T lymphocytes. SSC/CD3 was gated. (b) Intracellular expression of IL-4 on CD3^+^CD4^+^ T cells. (c) Intracellular expression of IFN-*γ* on CD3^+^CD4^+^ T cells. (d) Percentage of Th2 cells. (e) Percentage of Th1 cells. (f) Th2/Th1 ratio among healthy control, stable COPD, and AECOPD groups. Data are shown as mean ± SD; ^∗^*P* < 0.05, ^∗∗∗^*P* < 0.001, indicating comparison with the control group. ^###^*P* < 0.001 indicating comparison with the stable COPD group.

We used flow cytometry to detect the changes of Th1 and Th2 cells in peripheral blood of the AECOPD group, the stable COPD group, and the healthy group, respectively. CD3^+^ T lymphocytes were gated (Figure 1(a)). Compared with the control group and the stable COPD group, the proportion of peripheral blood Th2 (Figures 1(b) and 1(d)) and Th1 cells (Figures 1(c) and 1(e)) in CD4^+^ T cells was significantly increased in the AECOPD group (*P* < 0.001). At the same time, the AECOPD group had significantly higher proportion of peripheral blood Th2 and Th1 cells in CD4^+^ T cells than the stable COPD group (*P* < 0.001). However, the ratio of Th2/Th1 in the peripheral blood of the stable COPD group decreased, while that of the AECOPD group increased significantly than the control group (Figure 1(f)) (*P* < 0.001). The AECOPD group had significantly higher Th2/Th1 ratio than the stable COPD group (*P* < 0.001). These results suggest that there are different Th2/Th1 shifts in different courses of COPD, and in AECOPD, Th2/Th1 balance shifts to the Th2.

### 3.3. ILC2s in the Peripheral Blood of AECOPD Are Significantly Increased

**Figure 2:**
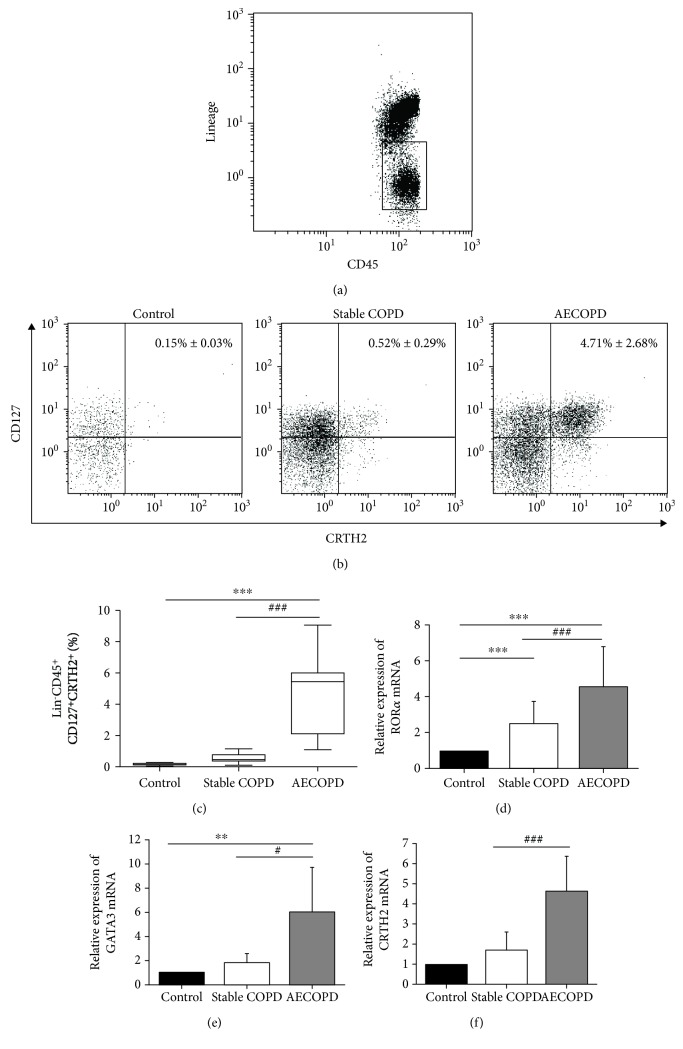
Levels of ILC2 and the related transcription factors of the three groups. Flow cytometry was performed. (a–c) Percentage of ILC2s in the peripheral blood of control, stable COPD group, and AECOPD group. The mRNA levels of (d) ROR*α*, (e) GATA3, and (f) CRTH2 were tested by RT-PCR. Data were normalized to control group and represented as mean ± SD. Compared with the control group, ^∗∗^*P* < 0.01, ^∗∗∗^*P* < 0.001. Compared with the stable COPD group, ^#^*P* < 0.05, ^###^*P* < 0.001.

In this study, we followed the strategy used by Mjosberg et al. [23] to identify ILC2s. We used lineage antibodies, including anti-CD3, CD19, CD123, CD11b, CD11c, CD8, CD14, CD4, CD56, and Fc*ε*RI*α* to identify T cells, monocytes, neutrophils, B cells, NK cells, mast cells, and basophils. Lin^−^ cells, which are negative for these lineage markers, were gated. ILC2s were expressed as Lineage^−^CD45^+^CD127^+^CRTH2^+^ cells. The proportion of ILC2s in the peripheral blood of the AECOPD group, the stable COPD group, and the healthy group was detected by flow cytometry. Lineage^−^CD45^+^ cells are shown in Figure 2(a), and the Lineage^−^CD45^+^CD127^+^CRTH2^+^ cells are shown in Figure 2(b). The percentage of ILC2s in the peripheral blood of the AECOPD group (4.71% ± 2.68%) was significantly higher than that in the stable COPD group (0.52% ± 0.29%) and in the healthy group (0.15% ± 0.03%) (*P* < 0.001) (Figures 2(b) and 2(c)). Additionally, the AECOPD group had higher percentage of ILC2s than the stable COPD group (*P* < 0.001).

Studies [23–25] have shown that the transcription factors ROR*α* and GATA3 are important in the differentiation of ILC progenitor cells into ILC2s. By using real-time PCR, we measured the mRNA levels of these transcription factors in each group of ILC2s. The results showed that the levels of transcription factors ROR*α* and GATA3 mRNA in the AECOPD group were significantly higher than those in the healthy control group and the stable COPD group (*P* < 0.001, Figures 2(d) and 2(e)). ROR*α* and GATA3 mRNA levels in the COPD group were also significantly higher than those in the healthy control group (*P* < 0.001). Moreover, it is reported that after stimulation, ILC2s express high levels of CRTH2 to promote production of Th2-type cytokines [26]. Similarly, our results also showed that CRTH2 mRNA levels were significantly higher in the AECOPD group than in the stable COPD group and the healthy control group (*P* < 0.001, Figure 2(f)). The results indicated that ILC2s might be involved in the pathogenesis of COPD.

### 3.4. ILC2s in Patients with AECOPD Have High Expression of MHC II and CD80

**Figure 3:**
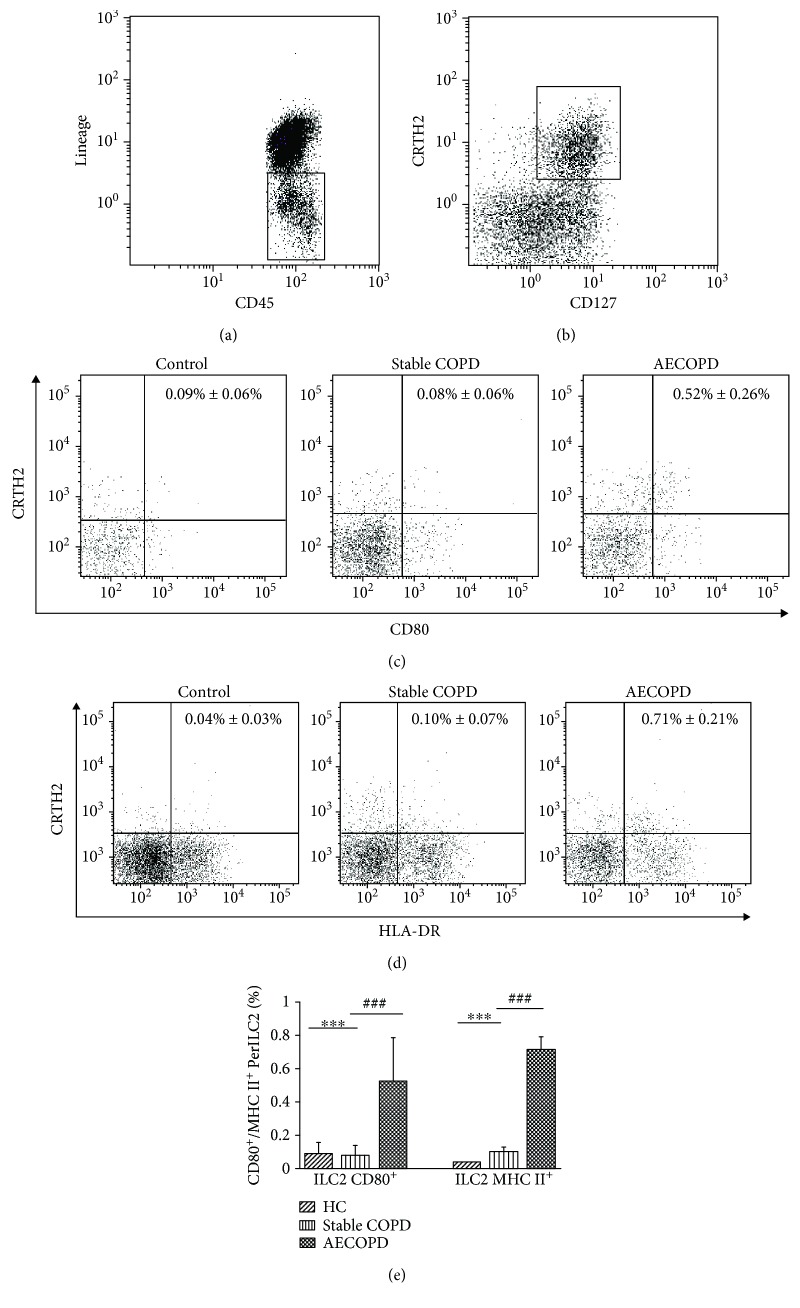
The level of MHC II and CD80 on ILC2s. Flow cytometry was performed. (a) Flow cytometric analysis of lineage-negative CD45^+^ cells is shown. (b) ILC2s are identified as Lin^−^CD45^+^CD127^+^CRTH2^+^. (c) CD80 and (d) MHC II on the surface of ILC2 in healthy control, stable COPD, and AECOPD groups. (e) The statistical results of percentage of CD80^+^ and MHC II^+^ within Lin^−^CD45^+^CD127^+^CRTH2^+^ cells in each group. Compared with the control group, ^∗∗∗^*P* < 0.001. Compared with the stable COPD group, ^###^*P* < 0.001.

By using flow cytometry, we further examined the expression of MHC II and CD80 on the surface of ILC2s in the AECOPD group, the stable COPD group, and the healthy control group, respectively (Figure 3). Lineage^−^CD45^+^ cells are shown in Figure 3(a), and the Lineage^−^CD45^+^CD127^+^CRTH2^+^ cells are shown in Figure 3(b). Compared with the healthy control group, the ILC2 CD80^+^ cells in the stable COPD group were slightly less but significantly increased in the AECOPD group (*P* < 0.001, Figures 3(c) and 3(e)). There was also a slight increase of MHC II^+^ ILC2 cells in the stable COPD group compared with the healthy control group, but there was no statistical significance. In contrast, the number of MHC II^+^ ILC2 cells in the AECOPD group increased significantly compared to the healthy control and stable COPD groups (*P* < 0.001, Figures 3(d) and 3(e)). The high expression of MHC class II molecules and costimulatory molecule CD80 on the surface of ILC2s in the AECOPD group suggests that ILC2s may participate in the immune modulatory process of AECOPD by affecting the function of CD4^+^ T cells.

### 3.5. Th2 Cells in the AECOPD Group Were Positively Correlated with MHC II^+^ ILC2 Cells

Subsequently, we analyzed the correlation between Th2 cells and ILC2s expressing CD80 and MHC II in the stable COPD group and the AECOPD group. Pearson correlation analysis showed that there was no significant correlation between Th2 cells and CD80^+^ ILC2 cells and MHC II^+^ ILC2 cells in the stable COPD group (Table 3). Similarly, Th2 cells in the AECOPD group were not significantly correlated with CD80^+^ ILC2 cells. However, there was a significant positive correlation between the ratio of Th2 cells and the ratio of MHC II^+^ ILC2 cells in the AECOPD group (*r* = 0.641, *P* = 0.018). The results indicated that MHC II^+^ ILC2 might promote Th2 immunity in AECOPD.

### 3.6. MHC II^+^ ILC2 Promotes Differentiation of Th2 Cells in AECOPD

**Figure 4:**
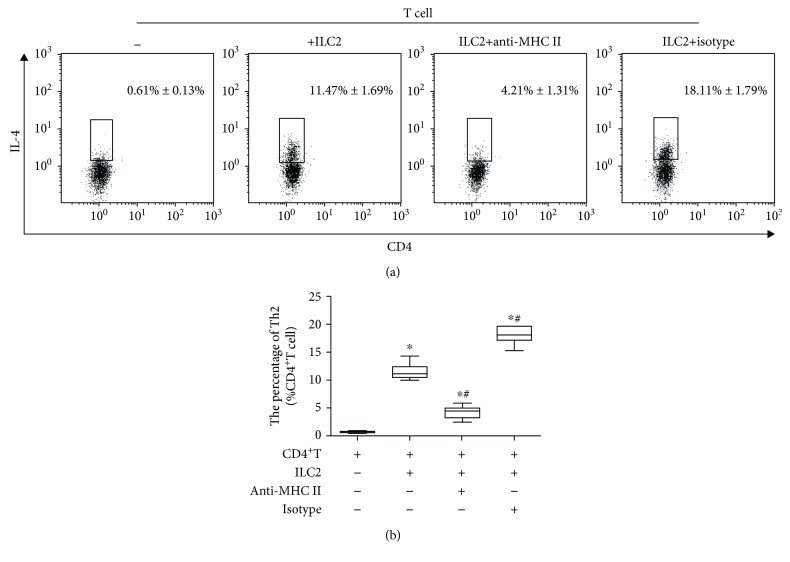
Level of Th2 cells after coculture with ILC2. Flow cytometry was performed. (a) Intracellular IL-4 staining following coculture of ILC2 and CD4^+^ T cell. (b) Percentage of Th2 cells. Data are shown as mean ± SD. Compared with the CD4^+^ T cell group, ^∗^*P* < 0.0. Compared with the ILC2 group, ^#^*P* < 0.01. Compared with the ILC2+anti-MHC II cell group, ^△^*P* < 0.01.

**Figure 5:**
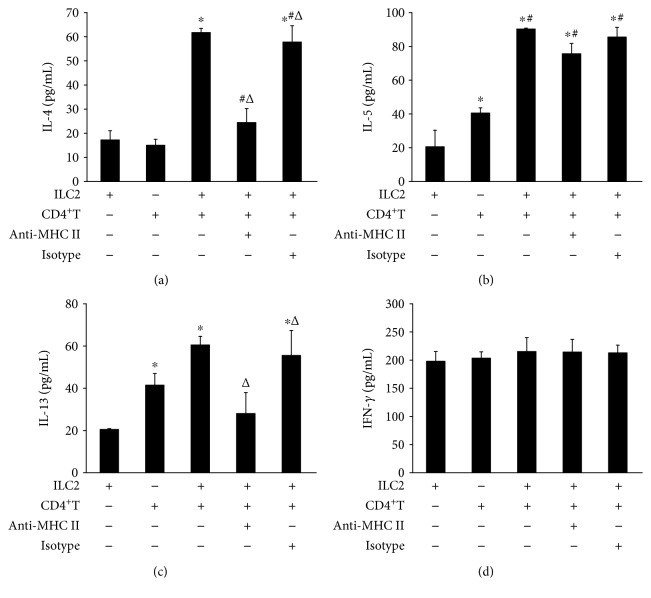
Cytokines in the supernatant of CD4^+^ T cell coculture with ILC2. (a) IL-4, (b) IL-5, (c) IL-13, and (d) IFN-*γ* in the coculture supernatant were tested by ELISA. Data were shown as mean ± SD. Compared with the ILC2 group, ^∗^*P* < 0.05; compared with the CD4^+^ T group, ^#^*P* < 0.01; compared with the ILC2:CD4^+^ T cell coculture group, ^△^*P* < 0.05.

To determine the function of ILC2 on the differentiation of Th2 cells, we sorted out ILC2s from the peripheral blood of the AECOPD group patients and CD4^+^ T cells from the peripheral blood of healthy donors using magnetic bead sorting. Then, we cocultured ILC2s with CD4 T cells at a 1 : 1 ratio. Flow cytometry result found that ILC2s derived from the AECOPD group promoted the differentiation of CD4^+^ T cells into Th2 cells (Figure 4). However, when we used the anti-MHC II neutralizing antibody to interfere with the interaction of MHC II with T cells, the number of Th2 cells decreased. At the same time, the ELISA results showed that IL-4, IL-13, and IL-5 were highly expressed in the culture supernatant after coculture of ILC2 with CD4 T cells (Figures 5(a)–5(c)). However, when anti-MHC II antibody was added, the levels of IL-4, IL-13, and IL-5 were significantly reduced. However, no significant difference in IFN-*γ* was observed in each group (Figure 5(d)). Through *in vitro* experiments, we confirmed that ILC2 of AECOPD could induce the differentiation of CD4^+^ T cells into Th2 by high expression of MHC II and promote the high expression of Th2-type cytokines, but its effect on Th1 cells was not significant. Therefore, MHC II^+^ ILC2 could promote the shift of Th2/Th1 balance to Th2 direction in AECOPD.

## 4. Discussion

The main pathological features of COPD are chronic inflammation of the airway mucosa [27]. Moreover, the T cell-mediated adaptive immune response is one of the important factors leading to the persistence and amplification of chronic inflammation of the lungs [28]. Study has demonstrated that the high inflammatory response of the COPD airway was mainly caused by the imbalance of Th cells [29]. According to the different cytokines, Th cells can be mainly divided into Th1 and Th2 cells. IFN-*γ* and IL-4 are the characteristic cytokines of Th1 and Th2 cells, respectively [30]. Normally, Th1/Th2 cells are in a relatively balanced state under normal conditions. However, studies have shown that there are imbalances of Th1/Th2 in different periods of COPD [10, 31]. In this study, we found that Th1 cells gradually increased in stable phase of COPD and AECOPD, and the expression of IFN-*γ* also showed the same trend. In contrast, the number of Th2 cells increased significantly, along with the serum IL-4 level. Meanwhile, the Th2/Th1 ratio was decreased in patients with stable COPD whereas it increased in patients with AECOPD, indicating that the Th2/Th1 balance is shifted to Th1 response in stable COPD while it shifted to Th2 response in AECOPD.

Studies [32–34] have shown that ILC2 is involved in the pathogenesis of COPD. We found that the levels of ILC2-associated transcription factors of ROR*α* mRNA, GATA3 mRNA, and CRTH2 mRNA were significantly increased in patients with stable COPD and were even higher in AECOPD patients. At the same time, flow cytometry also showed that ILC2s in the peripheral blood of the COPD group and the AECOPD group increased significantly, suggesting that ILC2s play a certain biological role in the process of COPD.

Many studies have shown the important role of ILC2s in Th2-type responses [18, 19]. Earlier, ILC2s were named as NHCs (natural helper cells) [35], nuocytes [13], or Ih2 [36]. This cell group differs from lymphoid progenitor or lymphoid tissue-inducing cells and does not express Lineage (Lin) marker. However, it expresses c-Kit, Sca-1, IL-7R, and IL-33R (T1/ST2) [37]. Like Th2 cells, ILC2s secrete IL-5 and IL-13 and participate in processes such as antihelminth infection [38], tissue repair after influenza virus infection [39], and allergic asthma [40]. Similar to Th2 cells, ILC2s are widely found in the intestine, mesenteric lymph nodes, lungs, lung draining lymph nodes, trachea, spleen, liver, Peyer's knot, skin, animal fat, and blood [41]. A large number of studies [42, 43] have shown that ILC2s can rapidly secrete Th2-type cytokines and are the main source of Th2-type cytokines. Moreover, studies have suggested an increased infiltration of ILC2s in tissues from patients with chronic type 2 inflammation, such as those with atopic dermatitis and nasal polyps [44, 45]. ILC2s might also play a previously unanticipated role in the initiation of Th2-type immunity.

Von Burg et al. found that ILC2s not only played an important role in early innate immunity but could also regulate adaptive cellular immunity by changing the direction of CD4^+^ T cell differentiation [20]. Cytokines are one of the factors for the regulation of Th2 differentiation, and IL-2 is critical for the proliferation of Th2 cells in the presence of ILC2s. IL-2 is expressed by T cells following CD28 ligation [46, 47] and plays an important role in Th2 differentiation via STAT5a signaling [48]. Notably, we found that the CD28 ligand CD80 was highly expressed on ILC2 of AECOPD patients, suggesting that ILC2s have the capacity to interact with T cells and contribute to their type 2 polarization.

Studies have shown that MHC class II molecules on APC are also involved in the regulation of the differentiation process of Th2 cells [49, 50]. Moreover, studies [18, 51] have found that ILC2s also express MHC class II molecules and are involved in the regulation of Th2 cell differentiation. Here, we found that ILC2 in patients with stable COPD expressed low levels of MHC II. However, the level of MHC II on the surface of ILC2s was significantly elevated in AECOPD patients, and its expression level was positively correlated with the number of Th2 cells. Therefore, we believe that in AECOPD, ILC2s with high expression of MHC II are likely to play a similar APC function and interact with T cell receptor through MHC class II molecules to promote the differentiation of Th2 cells, eventually leading to AECOPD Th1/Th2 balance shift to Th2 responses. The results of *in vitro* coculture of ILC2 and CD4^+^ T cells further showed that high expression of MHC II in AECOPD ILC2s promoted the differentiation of Th2 cells and the expression of Th2 cytokines. Moreover, this effect was inhibited after blocking with anti-MHC II antibodies. At the same time, in this study, we did not observe the differentiation of CD4^+^ cells into Th1 cells by MHC class II molecules. Thus, ILC2s can interact with CD4^+^ T cells, providing new insights into the underlying mechanisms of Th2-type immune-mediated diseases.

## 5. Conclusions

In summary, by highly expressing MHC II, ILC2 may function as APC, promoting Th2/Th1 balance to Th2 shift in AECOPD patients. Therefore, MHC II^+^ ILC2 may be a new target for clinical immunoassay and treatment of AECOPD. However, the underlying mechanism of ILC2 in COPD inflammatory response and pathological processes remains unclear and needs to be further studied.

## Figures and Tables

**Table 1:** The clinical data of the study cohort.

Factors	Healthy control (*n* = 110)	Stable COPD (*n* = 112)	AECOPD (*n* = 98)	*P* value^∗^	*P* value^∗∗^	*P* value^∗∗∗^
Age	66 ± 6.9	67 ± 8.2	67 ± 8.5	0.45	0.18	0.64
Gender *n* (%)						
Male	69 (62.7)	68 (60.7)	59 (60.2)	>0.05	>0.05	>0.05
Female	41 (37.3)	44 (39.3)	39 (39.8)	>0.05	>0.05	>0.05
Stages	—	16.4 ± 15.04	17.3 ± 12.3		>0.05	>0.05
History of smoking *n* (%)						
Yes	78 (69.6)	76 (67.9)	66 (67.3)	>0.05	>0.05	>0.05
No	32 (31.4)	36 (32.1)	31 (32.7)	>0.05	>0.05	>0.05
Pulmonary function						
FEV1/FVC	79 ± 5.18	61.5 ± 5.79	52.6 ± 7.5	<0.01	<0.01	<0.01
FEV1%	90.6 ± 12.9	65.38 ± 10.67	39.23 ± 7.8	<0.01	<0.01	<0.01
FVC%	96.07 ± 13.7	86.91 ± 15.09	60.5 ± 12.9	<0.01	<0.01	<0.01
Patient physical index						
BMI	24.75 ± 2.76	25.1 ± 3.4	24.5 ± 3.9	>0.05	>0.05	>0.05
6MWD (m)	416 ± 79	411 ± 71	392 ± 70	>0.05	>0.05	>0.05
mMRC	2 (1, 2)	2 (1, 2)	2 (2, 2)	>0.05	<0.05	>0.05
CAT	7 (4, 10)	9 (7, 14)	10 (6, 15)	>0.05	<0.05	<0.05
Inflammatory cytokines						
IL-4 (pg/mL)	29.71 ± 5.96	36.88 ± 3.05	55.16 ± 7.37	<0.5	<0.01	—
IFN-*γ* (pg/mL)	26.35 ± 4.67	27.97 ± 10.52	36.13 ± 11.80	<0.5	<0.01	—

AECOPD: acute exacerbations of chronic obstructive pulmonary disease; BMI: body mass index; COPD: chronic obstructive pulmonary disease; FEV1: forced expiratory volume in one second; FVC: forced vital capacity. FEV1/FVC was determined post bronchodilator. IL-4: interleukin-4; IFN-*γ*: interferon-*γ*; 6MWD: 6-minute walk distance; mMRC: Modified Medical Research Council Dyspnea Scale; CAT: Assessment Test Scale. Data were presented by mean ± SD, median (P25, P75), or percentage. The comparison between two groups was determined by *t-*test, Mann-Whitney *U* test, or chi-square test. A *P* < 0.05 was considered statically significant. ^∗^Comparison between AECOPD patients and stable COPD patients. ^∗∗^Comparison between AECOPD patients and HCs. ^∗∗∗^Comparison between stable COPD patients and HCs.

**Table 2:** Primers for RT-PCR.

Gene	Genbank accession	Forward primer	Reverse primer	Product size	Tm
		(5′→3′)	(5′→3′)	(bp)	(°C)
CRTH2	NM_004778.2	CGCCACACTGAAGCCACTCTG	GCGTGGTCGATGTAGCGGATG	90	60
RORA	NM_002943.3	CTGGTGTGCATAGCGGAGGTTG	CCTGCGGACTGGCAATAATCGG	101	60
GATA3	NM_001002295.1	GTGCATGACTCACTGGAGGACTTC	CATGTGGCTGGAGTGGCTGAAG	114	60
GAPDH	NM_002046	CAGGAGGCATTGCTGATGAT	GAAGGCTGGGGCTCATTT	138	60

**Table 3:** The correlations between Th2, ILC2 CD80^+^ cells, and ILC2 MHC II^+^ cells.

Th2 cell		ILC2 CD80^+^ cell	ILC2 MHC II^+^ cell
Stable COPD	*r*	0.126	0.439
	*P*	0.694	0.133
AECOPD	*r*	0.482	0.641
	*P*	0.095	0.018

## Data Availability

The data used to support the findings of this study are available from the corresponding authors upon request.
